# Machine learning to determine the main factors affecting creep rates in laser powder bed fusion

**DOI:** 10.1007/s10845-021-01785-0

**Published:** 2021-05-25

**Authors:** Salomé Sanchez, Divish Rengasamy, Christopher J. Hyde, Grazziela P. Figueredo, Benjamin Rothwell

**Affiliations:** 1 Faculty of Engineering, University of Nottingham, University Park, Nottingham, NG7 2RD UK; 2 School of Computer Science, University of Nottingham, Wollaton Rd, Lenton, Nottingham, NG8 1BB UK

**Keywords:** Machine learning, Creep, Additive manufacturing, Nickel superalloy, Predictability, Process–structure–property relationship

## Abstract

There is an increasing need for the use of additive manufacturing (AM) to produce improved critical application engineering components. However, the materials manufactured using AM perform well below their traditionally manufactured counterparts, particularly for creep and fatigue. Research has shown that this difference in performance is due to the complex relationships between AM process parameters which affect the material microstructure and consequently the mechanical performance as well. Therefore, it is necessary to understand the impact of different AM build parameters on the mechanical performance of parts. Machine learning (ML) models are able to find hidden relationships in data using iterative statistical analyses and have the potential to develop process–structure–property–performance relationships for manufacturing processes, including AM. The aim of this work is to apply ML techniques to materials testing data in order to understand the effect of AM process parameters on the creep rate of additively built nickel-based superalloy and to predict the creep rate of the material from these process parameters. In this work, the predictive capabilities of ML and its ability to develop process–structure–property relationships are applied to the creep properties of laser powder bed fused alloy 718. The input data for the ML model included the Laser Powder Bed Fusion (LPBF) build parameters used—build orientation, scan strategy and number of lasers—and geometrical material descriptors which were extracted from optical microscope porosity images using image analysis techniques. The ML model was used to predict the minimum creep rate of the Laser Powder Bed Fused alloy 718 samples, which had been creep tested at $650\,^\circ$C and 600 MPa. The ML model was also used to identify the most relevant material descriptors affecting the minimum creep rate of the material (determined by using an ensemble feature importance framework). The creep rate was accurately predicted with a percentage error of $1.40\%$ in the best case. The most important material descriptors were found to be part density, number of pores, build orientation and scan strategy. These findings show the applicability and potential of using ML to determine and predict the mechanical properties of materials fabricated via different manufacturing processes, and to find process–structure–property relationships in AM. This increases the readiness of AM for use in critical applications.

## Introduction

In applications such as aerospace jet engines, some components (e.g. first stage turbine discs/blades) operate under extreme temperatures and stresses. These components are critical and therefore cannot fail in service or enter service in a faulty condition (Chua 2017). Turbine discs, for example, must be manufactured from materials with adequate mechanical properties, such as high fatigue and creep resistance, strength and mechanical integrity at elevated temperatures (Ashby and Jones 2012). In particular, creep is one of the most significant causes of failure of such components as temperatures increase (Reed 2006). Nickel-based superalloys currently offer the best creep resistance, strength at high temperatures and cost balance compared to other metal alloys. Currently, nickel-based superalloy turbine discs are produced by using conventional subtractive manufacturing methods, which restrict component design, leading to sub-optimal efficiency. Various conventional manufacturing methods result in different properties, for example, casting results in larger grains and better creep performance compared to wrought material (Geddes 2010). Additive manufacturing (AM) could allow innovative designs, such as internal cooling channels, to easily be integrated and manufactured at no extra cost or time (Babu et al. 2018). Laser Powder Bed Fusion (LPBF) is one of the main AM processes used for metal manufacturing and nickel-based superalloys, particularly alloy 718, have some of the best current LPBF printability (Carter 2013).

Alloy 718 is composed of a matrix phase, $\gamma$ and strengthening phases like $\gamma '$ ($Ni_3(Al Ti)$) and $\gamma ''$ ($Ni_3 Nb$), which are precipitated during heat treatment (Reed 2006). Some detrimental phases include the Laves phase, which embrittles grain boundaries and negatively impacts creep properties (Chlebus et al. 2015), carbides and TCP phases. Other phases such as the $\delta$ phase have been stated as both beneficial (Balachandramurthi et al. 2019) and detrimental (Geddes 2010) to creep properties. The size, amount and location of these secondary precipitates determine the mechanical properties of the alloy. The LPBF process results in a specific anisotropic microstructure with elongated columnar grains along the build direction (BD) and equiaxed grains normal to the BD, due to complex cooling gradients present in the part. This results in anisotropic mechanical properties as well (Sabelkin et al. 2019). Furthermore, the mechanical performance of LPBF nicke-based superalloys is not yet fully understood and results in lower mechanical performance than conventionally manufactured materials (Xu et al. 2018).

For LPBF built components to be able to replace conventionally manufactured equivalents, it is of great importance to understand the material behaviour of LPBF built material and to be able to predict the mechanical performance. There are 3 main categories of creep material behaviour models for creep analysis (Hyde 2014). First, the Norton Power-law equations which describe the secondary creep behaviour. Then, damage mechanics models, like the Kachanov, Liu-Murakami and Dyson models, which can predict secondary creep, tertiary creep and failure times. As AM materials are notoriously porous, damage models could be applicable and useful to model the creep behaviour LPBF alloys. Finally, unified material behaviour models, like the Chaboche viscoplasticity equations, which represent rate dependent plasticity, stress relaxation and creep behaviour, are also available. Finite Element Analysis (FEA) is a commonly used computational method, which uses the material models described above, for modelling creep behaviour. It allows mechanisms and failure modes to be better understood. FEA has been used extensively to model nickel-based superalloys and components (Maharaj et al. 2012). Furthermore, FEA and numerical models have been used to model AM microstructure and its evolution (Nie et al. 2014; Tan et al. 2020), thermal history (Promoppatum et al. 2018), process parameter influence on grain morphology (Raghavan et al. 2016), meltpool morphology predictions in LPBF alloy 718 (Romano et al. 2016) and more. However, FEA can be complex, computationally expensive and over/under estimate stresses (Saberi et al. 2020).

An emerging modelling trend in AM is to use machine learning (ML) models (Qi et al. 2019; Koeppe et al. 2018; Wang et al. 2020; Sanchez et al. 2021c). Fundamentally, ML models operate on the principle of minimising predicted error iteratively using data. They have been shown to be accurate predictive tools, as demonstrated by Shen et al. (2020) who succeeded in predicting tool wear using ML techniques, and Xia et al. (2021) who modelled and predicted the surface roughness of Wire Arc Additively Manufactured metal. ML is also gaining attention as a process monitoring and control tool for AM, as demonstrated by Li et al. (2020) and Baturynska and Martinsen (2021). A review by Wang et al. (2020) on the use of ML in AM shows that most of the studies used process parameters (such as laser power, scan speed and build orientation) as inputs and both material and mechanical properties like porosity, hardness and fatigue resistance, as outputs. This was also highlighted in a review on the use of Neural-Network-based ML in AM where Fused Deposition Modelling, Selective Laser Sintering, Binder Jetting and Electron Beam Melting used build parameters in a ML model to predict the density, build time, tensile strength, dimensional accuracy and more (Qi et al. 2019). Both reviews underline the potential of using ML to establish process–structure–property–performance relationships in AM, without the need for underlying physical models linkage (Wang et al. 2020; Qi et al. 2019). However, one of the key limitations of using ML with LPBF is the small data set available to train the models (Wang et al. 2020). But this can be overcome by using data augmentation (Wang et al. 2020; Wong et al. 2016).There is currently a limited use of ML for LPBF process parameter optimisation (Wang et al. 2020) and few studies have conducted research on predicting mechanical properties from LPBF process parameters and material data (Qi et al. 2019) . Moreover, although establishing process–structure–property relationships has been highlighted as a key area of using ML with AM, there are very few studies which actually used ML to determine the effect of AM process parameters on the materials’ microstructure and mechanical properties. There is therefore a gap in research which this study hopes to fill by using ML models to predict the creep rate of LPBF alloy 718 and determine the main process parameters affecting the creep rate.

From the literature it is clear that there is currently limited use of ML to predict and understand the relationship between LPBF build parameters and mechanical performance. Furthermore, LPBF specific models for predicting creep rate or creep life are scarce. It is therefore necessary to evaluate the potential of using a ML method within an interpretation framework which allows to understand the relationships between process parameters and to predict the resulting minimum creep rate. This will prove beneficial to deliver more targeted research and must be investigated if LPBF components are to be used in critical applications. Thus, investigating factors influencing creep rates with the help of ML models is timely, as it has the potential of identifying useful hidden patterns in a dataset. Within the present paper, for the first time, a ML model will be presented using LPBF build parameters and porosity data as inputs to predict the creep rate of alloy 718. In order to do this, LPBF samples will be built using different process parameters (build orientation, scan strategy, number of lasers) and porosity data will be taken from these samples before creep testing. The porosity data and minimum creep rate obtained will be used to extract additional material descriptors to be used in different ML models. ML models to be tested include, Random Forest (RF), Gradient Boosted Tree (GBT), Support Vector Regressor (SVR), Deep Neural Network (DNN), Ridge Regressor, and Least Absolute Shrinkage and Selection Operator (LASSO) Regressor. Subsequently, the material descriptor importance will be quantified using RF, GBT, and SVR through ensemble feature importance (Rengasamy et al. 2020) and then reported and discussed along with the result of creep rate prediction.

## Methodology

**Fig. 1:**
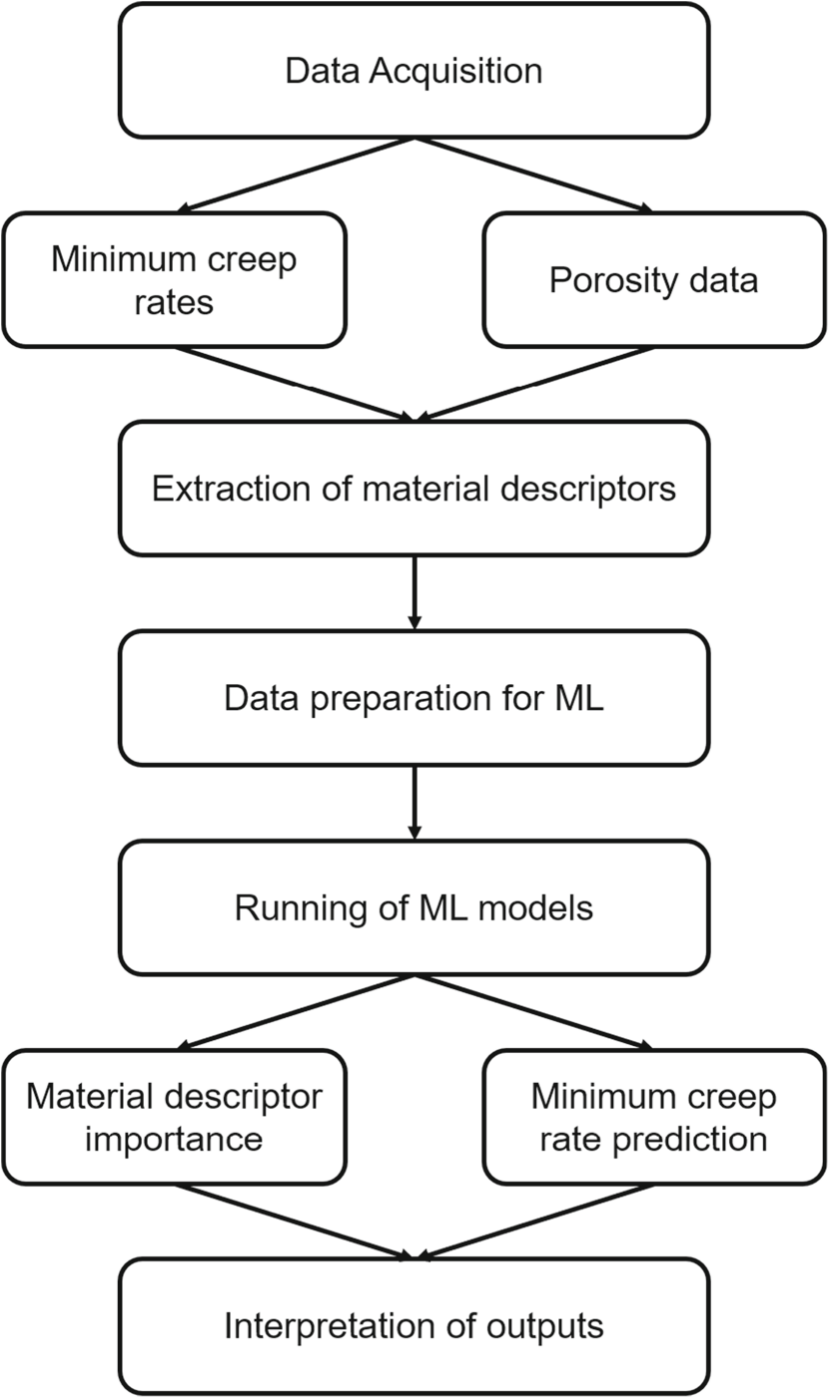
Flowchart of the methodology followed in this work. First the minimum creep rates and porosity data were obtained during the Data Acquisition phase, Then Material descriptors were extracted before preparing all of the available data for the ML models. Next, different ML models were ran. They predicted the minimum creep rate of the material and identified the main parameters which affected the minimum creep rate. Finally the model outputs were interpreted to verify and understand the findings

In order to predict the creep rate of AM alloy 718 material using different ML models, experimental data were first acquired by creep testing LPBF alloy 718 samples and performing a porosity analysis on non-creep tested samples. Subsequently, data from the samples was used to generate material descriptors. Generated material descriptors and test cases were concatenated to produce the final data for ML models to predict creep rate. Finally, ensemble material descriptor importance (Rengasamy et al. 2020) was employed to study the effect of material descriptors on creep rate prediction. Figure 1 summarises the processes undertaken in this work.

### Data acquisition

To acquire input data for the ML models, creep data and porosity data were obtained from LPBF alloy 718 samples through mechanical testing and microscopy observations, respectively.

#### Creep data

Data was collected from creep tests, using the ASTM E139 standard, at a temperature of 650 $^\circ$C and under a stress of 600 MPa on a Denison constant load creep machine (T45A3), which outputs time and extensometer voltage (1 V = 1 mm). The objective of the tests was to obtain the minimum creep rates of LPBF alloy 718 built with several different AM build parameters. The specific parameters investigated are as follows:

- building orientation: 0$^\circ$ , 45$^\circ$ and 90$^\circ$;
- scan strategy: stripe or meander;
- number of lasers: 1 or 4 lasers.

**Fig. 2:**
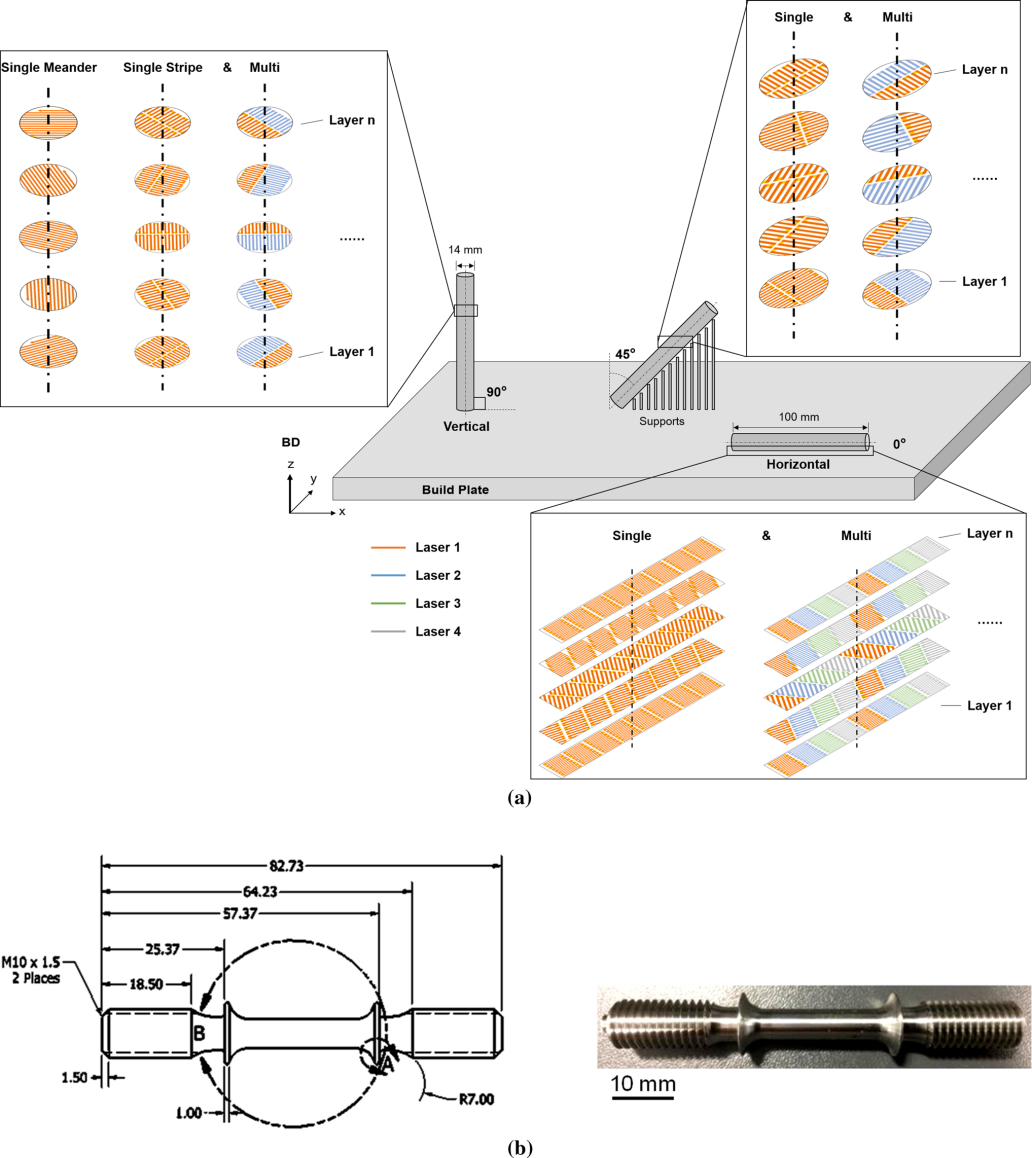
**a** Overview of the different parameters investigated: build orientation, scan strategy and number of lasers; **b** ASTM E8/E8M (ASTM 2016) uniaxial sample after turning and grinding

The different test cases—Vertical Single laser Meander (VSM), Vertical Single laser Stripe (VS), Vertical Multi laser (VM), 45$^\circ$ Single laser (45S),45$^\circ$ Multi laser (45M), Horizontal Single laser (HS), and Horizontal Multi laser (HM)—along with their process parameters are shown in Table 1 and Fig. 2a shows a schematic of these different build strategies. Each test case had 3 repeats.

**Table 1:** Summary of the different test cases and their process parameters

Test case	Orientation	Number of lasers	Scan strategy	Power (W)	Stripe point distance ($\mu$m)	Hatch time ($\mu$s)	Exposure distance (mm)	Hatch offset (mm)
VSM	$90^\circ$	1	Meander	212.5	17	20	0.09	0
VS	$90^\circ$	1	Stripe	212.5	17	20	0.09	$-\,0.2$
VM	$90^\circ$	4	Stripe	212.5	17	20	0.09	$-\,0.2$
45S	$45^\circ$	1	Stripe	212.5	17	20	0.09	$-\,0.2$
45M	$45^\circ$	4	Stripe	212.5	17	20	0.09	$-\,0.2$
HS	$0^\circ$	1	Stripe	212.5	17	20	0.09	$-\,0.4$
HM	$0^\circ$	4	Stripe	212.5	17	20	0.09	$-\,0.4$

To achieve this, cylinders were built on a Renishaw 500Q using Renishaw alloy 718 powder, whose composition is shown in Table 2.

The samples were then heat treated directly on the build plate according to the AMS5662 standard (Alloy 1965) (980 $^\circ$C/1h/Gas quench, 720 $^\circ$C/8h/ Furnace Cooling to 620 $^\circ$C/8h/Gas quench). Following that, the samples were Wire Electric Discharge Machined from the build plate, then turned using a Tormach Slant-Pro slant lathe to ASTM E8/E8M standard (ASTM 2016) dimensions, which included knife edges used to attach extensometers during the creep tests. Finally, the sample gauge length was ground by using a P46 grit aluminium oxide grinding wheel mounted on a Jones and Shipman 1302 cylindrical grinder to obtain a better surface finish, which affects creep life. The final sample dimensions can be found in Fig. 2b.

#### Porosity data

**Fig. 3:**
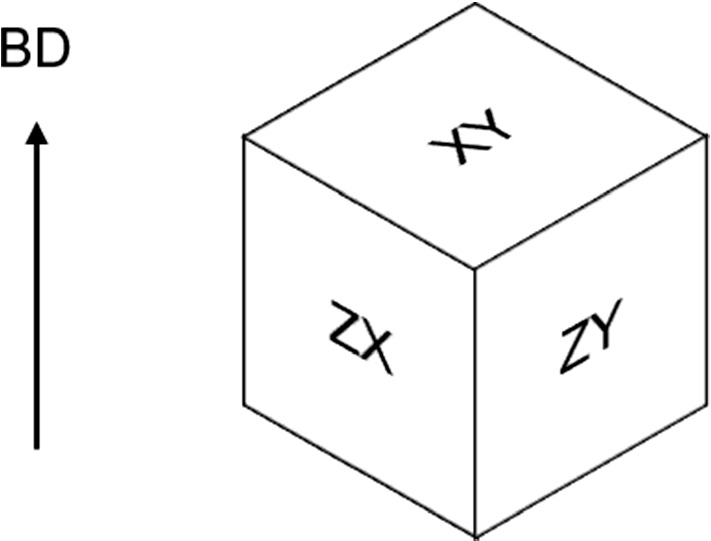
Cube planes used for microscopy analysis with respect to the Build Direction (BD)

As the aim of this study is to understand the effect of the LPBF parameters and pre-creep density, porosity analysis was conducted using a NIKON ECLIPSE LV100ND Optical microscope. Porosity analysis for the different test cases was done on non-creep-tested cubes which were cut in 3 perpendicular planes (Fig. 3)—except for 45S and 45M samples which were cut in 2 perpendicular planes—with a silicon carbide disc. 15 pictures were taken for each plane of each test case—except for 45S and 45M samples where only 10 images were extracted, resulting in 265 porosity images (5 test cases x 3 planes x 15 pictures + 2 test cases x 2 planes x 10 pictures = 265 images). Cubes ($10~mm^{3}$) were used instead of cylindrical creep samples for analysis purposes in order to save material. This is a common practice in AM research.

**Fig. 4:**
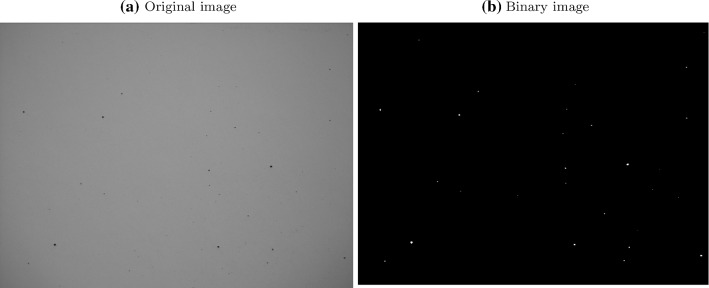
**a** Original image obtained from printed nickel-based superalloy cube with porosity shown by dark spots. **b** Black and white image with porosity labelled as white pixel using CCL

The porosities of each sample (see Table 1) on cube samples were identified through a series of image analysis steps. First, the images were converted to greyscale to remove the RGB channels. Second, a binary filter was applied to the greyscale image to differentiate the pores and non-pores pixels. Finally, a Connected-component Labelling (CCL) algorithm (Rosenfeld and Pfaltz 1966) was used to calculate the number of pores. CCL is a region labelling algorithm that scans an image and groups pixels of similar intensity value into a region based on pixel connectivity. Each connected group are subsequently assigned a label. The number of labels is equivalent to the number of pores in a single image. A binary or grey-scaled image is required to perform CCL. The original cube sample image, as shown in Fig. 4a was converted into a grey-scaled image by using the average of Red, Green, Blue pixel values. Subsequently, the grey-scaled image was converted into a binary image using a predetermined threshold value. Pixel values less than or equal to the threshold were set to a value of 1 while pixel values higher than the threshold were set as 0 to produce a binary image. The threshold value is the pixel value that ranges from 0 (black) to 255 (white). The sensitivity of this threshold is approximately 5. Any values higher or lower than 5 away from the threshold significantly affects the final count of porosity. The optimum threshold value was found by trial-and-error. The pores were denoted using the pixel value of 1 and the non-porous section with a value of 0, as shown in Fig. 4b. The number of pores was then identified using CCL from the binary image.

The CCL algorithm returned a unique number for each of the connected white region in the binary image.

### Extraction of material descriptors

Material descriptor engineering was performed on the porosity data to extract new geometrical and mathematical material descriptors. The new material descriptors were extracted using the *Python scikit-image* (Van Der Walt et al. 2014) library. The *scikit-image* library measured various properties for each connected region labelled in the binary images, more specifically the *regionprops* function under the *measure* modules. The new material descriptors generated contain information regarding the geometrical shape and mathematical properties, as shown in Table 3, along with a description the new material descriptors.

The material descriptors in Table 3 along with scan strategy, the number of lasers and build orientation were used to train ML models.

### Data preparation for machine learning models

**Fig. 5:**
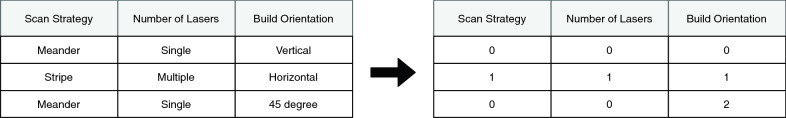
Transforming categorical data to numerical values using label encoding method

Data preparation was conducted to ensure that the data was suitable to be trained by the ML models. First, the categorical data was transformed into numerical data using the label encoding method (Seger 2018), as shown in Fig. 5.

**Table 2:** Composition of alloy 718 powder

Element	Weight %	Element	Weight %
Ni	52.5	Co	0.04
Cr	19.1	C	0.03
Fe	Bal.	Mn	0.02
Nb + Ta	4.89	N	0.01
Mo	3.2	Cu	0.01
Ti	0.86	P	$<0.01$
Al	0.42	S	0.001
Si	0.04	B	$<0.001$

Subsequently, the continuous material descriptors were scaled to [0, 1] range as shown in Equation 1 below:

1 $$\begin{aligned} z_{i} = \frac{x_{i} - min(x)}{max(x) - min(x)} \end{aligned}$$

Where *z* is the newly scaled material descriptors and *x* is the unscaled material descriptors. The material descriptors were scaled to ensure equal weighting is given to all material descriptors to prevent material descriptor bias in training. Next, the dataset was split into data instances (each data instance consists of material descriptors from one porosity image) into a 90/10 ratio that consisted of all samples. The 265 samples (porosity images) were cropped approxiamately in half to produce 512 samples as part of the data augmentation. Data augmentation is used to increase the amount of data by modifying existing data. Data augmentation also double as a regulariser for ML models to prevent overfitting (Shorten and Khoshgoftaar 2019). The structure of this data is to investigate the predictability of creep rate when all test cases were included in both training and test data. The ML models were first trained on the first nine subsets of data and tested on the last subset of data. In the subsequent iteration, ML models were trained on the first eight and the last subset of data and tested on the ninth subset data. The cycle continued until it finally tested the first subset of data. The results obtained from each iteration were collected, and the final result was then averaged across all ten iterations.

**Fig. 6:**
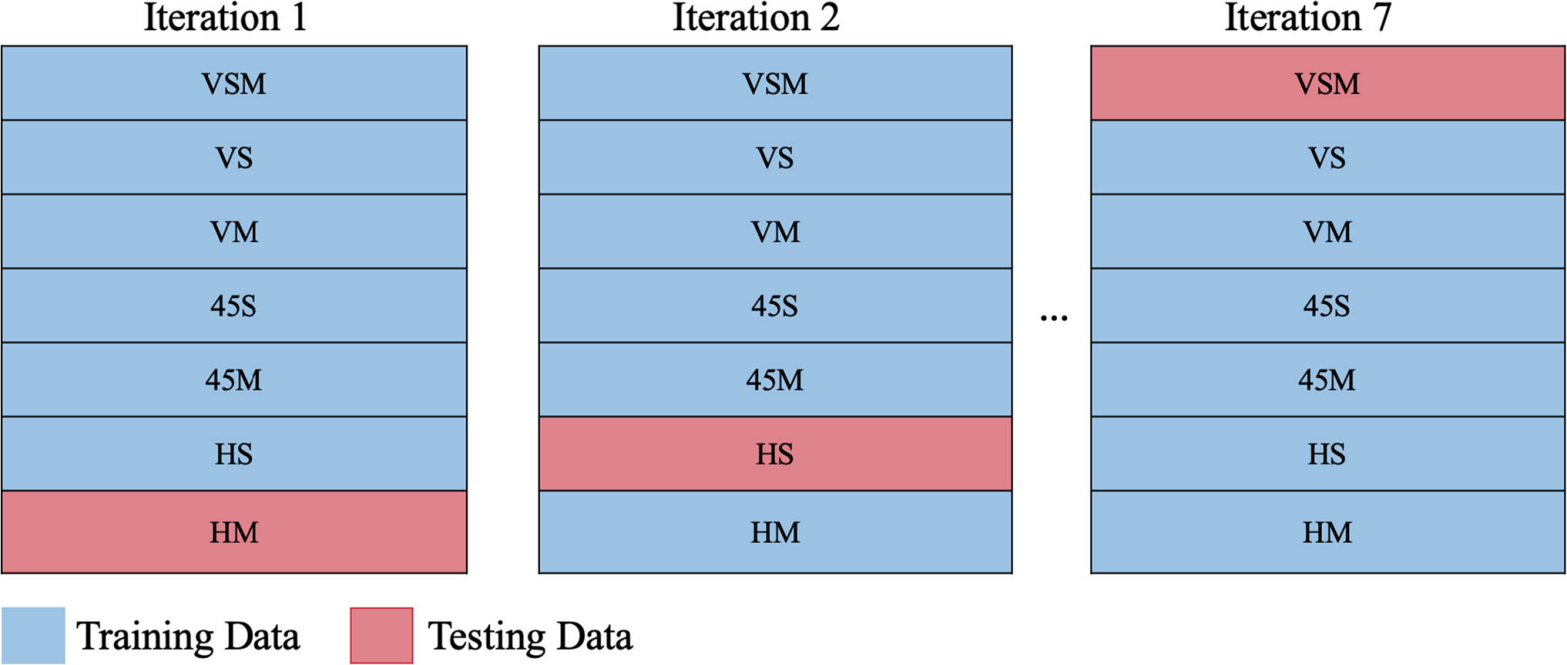
Creep rate prediction of ML models when one test case was excluded from the training data. The excluded test case was used as the testing data. Testing is repeated for each test case

Furthermore, Leave-One-Case-Out (LOCO) was set up to investigate the ML models creep rate prediction accuracy when it is trained in the absence of one unseen sample. LOCO is the direct application of Leave-One-Out cross validation where a subset of data is left out to be tested while the remaining is used to train the model. LOCO is designed to investigate if ML can accurately predict the creep rate of unseen test cases. For example, the ML models were first trained on data with the samples of VSM, VS, VM, 45S, 45M, and HS and then tested on HM data. The cycle continued for all test cases, as shown in Fig. 6.

**Table 3:** Material descriptors extracted from porosity images used to train ML models for secondary creep rate prediction using *scikit-image* (Van Der Walt et al. 2014)

Material descriptor	Description
Number of pores	The number of pores in the labelled image
Area	Number of pixels in the connected labelled region
Convex area	Convex hull image of labelled pixels, i.e. the smallest convex polygon that encloses the area
Eccentricity	Eccentricity of the ellipse that has the same second-moments as the labelled area. The eccentricity is the ratio of the focal distance (distance between focal points) over the major axis length. The value is in the interval of 0 and 1. When it is 0, the ellipse becomes a circle
Equivalent diameter	The diameter of a circle with the same area as the region
Major axis length	Major axis length of the ellipse
Minor axis length	Minor axis length of the ellipse
Orientation	Angle between the row axis of image and the major axis of the ellipse that has the same second moments as the region, ranging from -pi/2 to pi/2 counter-clockwise
Perimeter	Perimeter of object which approximates the contour as a line through the centers of border pixels using a 4-connectivity
Density (solidity)	Ratio of pixels in the area to pixels of the convex hull image
Inertia tensor	Inertia tensor of the area for the rotation around its mass
Inertia tensor eigenvalues	The eigenvalues of the inertia tensor in decreasing order

### Machine learning models

The ML models selected for both experiments were RF, GBT, DNN, SVR, Ridge Regressor, and LASSO Regressor. Regularised linear models such as Ridge and LASSO Regressors were chosen for their simplicity. RF and GBT were selected as both models have shown great results for structured and tabular data (Chen and Guestrin 2016). Furthermore, DNN (Qi et al. 2019; Zhang et al. 2019; Francis and Bian 2019) and SVR (Song et al. 2016; Li et al. 2019) were also investigated as several AM papers have shown great result using these methods. All ML models were implemented using *scikit-learn* (Pedregosa et al. 2011) except for DNN, which were implemented using Tensorflow (Abadi et al. 2015).

#### Regularised linear regression

Linear regression is a simple statistical learning approach to map a vector of predictor variable, ${\overline{X}}$, to a quantitative target value, *Y*. The relationship between ${\overline{X}}$ and *Y* is represented through its coefficients, ${\overline{\beta }}$, and an intercept value, $\alpha$. The linear regression formula can be represented as follow:

2 $$\begin{aligned} Y = {\overline{\beta }}\cdot {\overline{X}} + \alpha \end{aligned}$$

Subsequently, the linear regression model learn the coefficient values by minimising the error based on the least square criterion:

3 $$\begin{aligned} min(Y - ({\overline{\beta }}\cdot {\overline{X}} + \alpha ))^{2} \end{aligned}$$

Once the coefficients are determined, it can generate a prediction of target value, ${\hat{Y}}$. One way of calculating the error of this prediction is the squared error (SE):

4 $$\begin{aligned} SE = (Y - {\hat{Y}})^{2} \end{aligned}$$

A trained model can be overly complex and thus overfit the data. A strategy to discourage this behaviour is to penalise the magnitude of the coefficients in addition to the prediction error. The two commonly used regularisation terms are Ridge regression and LASSO regression. The Ridge and LASSO regression perform the sum of square of the coefficients, and the sum ofthe absolute value of the coefficients, respectively. The new goal is to minimise the prediction error, and the regularisation terms as follow:

5 $$\begin{aligned}&\text {Ridge Regression}: min \left( SE + \sum _{n=1}^{N} \lambda \beta _{n}^{2}\right) \end{aligned}$$

6 $$\begin{aligned}&\quad \text {LASSO Regression}: min\left( SE + \sum _{n=1}^{N} \lambda |\beta _{n}|\right) \end{aligned}$$

The $\lambda$ term controls the regularisation strength. As $\lambda \rightarrow 0$ the values of $\beta$ also approaches 0. A Ridge regression pushes the non-importance coefficients close to zero while LASSO sets them as zero. Therefore, Ridge regression is more suitable if all predictors are necessary while LASSO regression is better for eliminating non-important predictors. The value of $\lambda$ for Ridge and LASSO Regressors is set to 1.0 for this paper.

#### Random forest

**Fig. 7:**
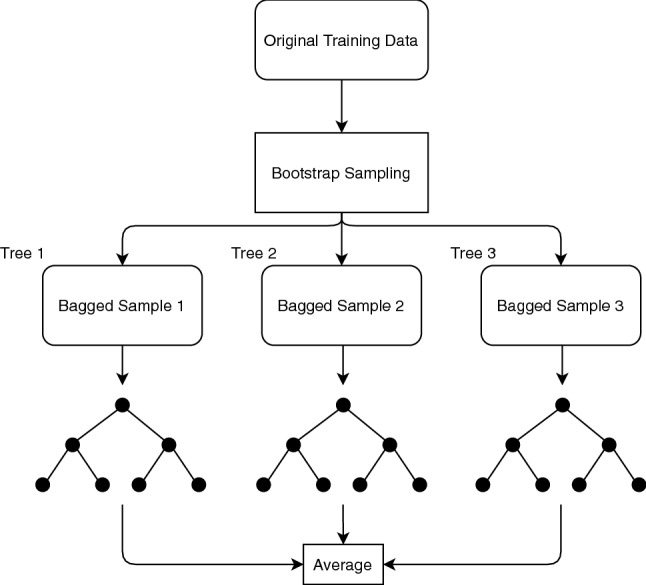
Schematic representation of the Random Forest model

RF is a tree-based learner based on the idea of bootstrap aggregating (bagging). The first step of RF is bootstrap sample. Bootstrap sampling is a method to draw samples from data with replacement. The original dataset is resampled into multiple smaller datasets, as shown in Fig. 7. Each smaller subset of data has approximately $\sqrt{p}$ material descriptors, where *p* is the original number of material descriptors. Those subsets are trained by a decision tree, and their outputs are averaged to reduce bias and variance in the results.

The hyperparameters used for RF are in Table 4. Hyperparameters are configurations on the learning model used for training input data. Unreported hyperparameters used were the default value set in *scikit-learn*. All hyperparameters were optimised using a random search method. (Bergstra and Bengio 2012). Random search is an optimisation technique that is commonly employed in ML hyperparameters optimisation. A search space of pre-determined values for different hyperparameters is randomly sampled and used to train ML models. This process is repeated N number of times and the best performing model along with its hyperparameters is used for the actual training and testing process.

**Table 4:** Hyperparameters used to train random forest to predict creep rate

Hyperparameter	Value
Number of trees	500
Maximum material descriptors	$\sqrt{p}$
Maximum depth of trees	5 level
Minimum samples before split	2
Bootstrap	True

#### Gradient boosted tree

**Fig. 8:**
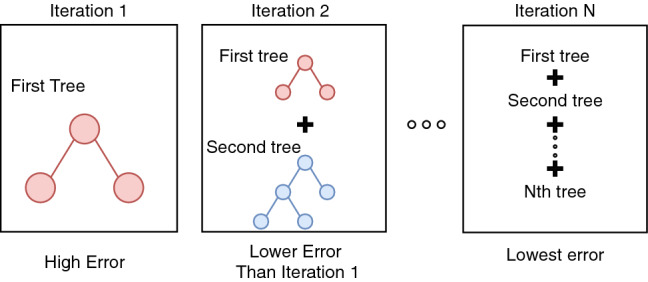
Schematic representation of the Gradient boosted tree model

Similar to RF, GBT is also a tree-based learner. GBT differs from RF in that it does not create multiple copies of decision trees and average the results from all trees. Instead, trees are grown sequentially by adding previously learned trees in each iteration until the minimal error is achieved as shown in Fig. 8.

The hyperparameters used for GBT are shown in Table 5. Unreported hyperparameters used were the default value set in *scikit-learn* (Pedregosa et al. 2011).

**Table 5:** Hyperparameters used to train Gradient Boosted Tree to predict creep rate

Hyperparameter	Value
Number of trees	500
Learning Rate	0.1
Maximum depth of trees	5 level
Loss function	Least square
Maximum material descriptors	$\sqrt{p}$
Splitting criterion	Friedman MSE

#### Support vector regression

SVR is an optimisation-based ML approach to solving regression problem. The goal of SVR is to find a function that can predict a target, *Y*, within a certain margin of error, $\epsilon$. This can be reformulated in optimisation terms as follow:

$$\begin{aligned}&min\left( \frac{1}{2}||\beta ||^{2}\right) \\&\quad s.t.\; Y - \beta X - \alpha \le \epsilon ; \\&\quad \beta X + \alpha - Y \le \epsilon ; \end{aligned}$$

where ${\beta {X}} + \alpha$ is the linear equation predicting the value *Y*. The minimisation of $\frac{1}{2}||\beta ||^{2}$ is to ensure the that the margin of boundaries within $\epsilon$ is as flat as possible. If the data (*X*, *Y*) is non-linear, the above formulation of SVR will not work. To overcome the issue of non-linearity for SVR, the data are mapped to a higher dimension using a kernel function. Non-linear can be converted to linear data with probability when it is mapped onto a higher dimensional space as shown by the Cover’s Theorem (Cover 1965). The hyperparameters of SVR used is in Table 6.

**Table 6:** Hyperparameters used to train Support Vector Regressor to predict creep rate

Hyperparameter	value
Kernel function	Radial Basis Function
Margin of error	0.1
Regularisation	1.0
Maximum Iteration	No limit

#### Deep neural network

**Fig. 9:**
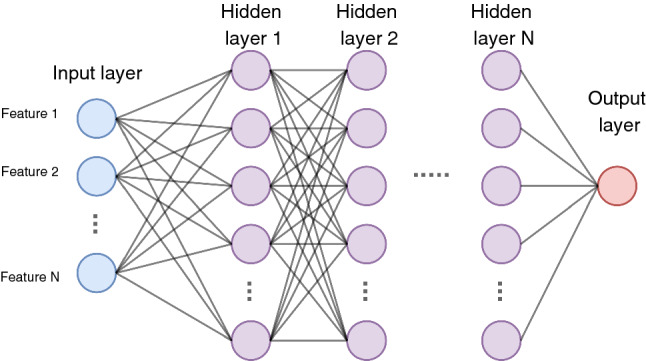
Structure of a deep neural network with 3 different layer types, namely the input layer, hidden layers and output layer

DNN is an extension of the neural network to a larger model size. DNN is structurally similar to the neural network where there are three types of layers, as shown in Fig. 9. A DNN can have any arbitrary number of hidden layers greater than one, with each layer consisting of one or more nodes. The input layer uses the training data and each node on the input layer accepts a material descriptor of the input data. The hidden layers are where most of the network’s parameters — weights and biases are located. The hidden layers are also responsible for most of the data transformation. A deep neural network has a more extensive number of hidden layers compared to a neural network. Additionally, the output layer is where the data are transformed to a predefined output data type. The parameters of the network were optimised using the Backpropagation algorithms (Chauvin and Rumelhart 1995) during training.

The hyperparameters of DNN used for both experiments ares in Table 7. The number of hidden layers and number of nodes for each layer were selected based on trial-and-error. The number of layers attempted ranges from 2 to 10 and the number of neurons for each layer attempted ranges from 4 to 256. More complex automated hyperparameter tuning methods, such as genetic algorithms and bayesian optimisation, were not used as the DNN employed in this paper is relatively small.

**Table 7:** Hyperparameters used to train Deep Neural Network to predict creep rate

Hyperparameter	Value
Number of hidden layers	8
Number of nodes for each layer	256, 128, 64, 32, 16, 8, 6, 4
Activation function	Rectified Linear Unit
Loss function	MSE
Optimiser	Rectified Adam with LookAhead (Tong et al. 2019)
Learning rate	0.001
L2 regulariser	0.01

**Fig. 10:**
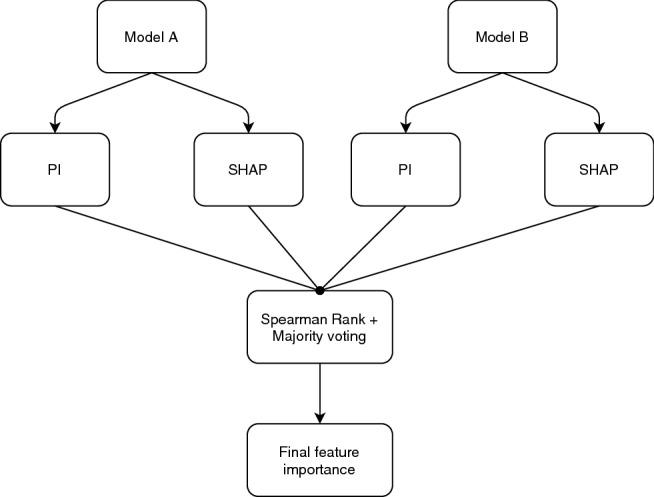
The permutation importance (PI) and SHapely Additive exPlanation (SHAP) from ML model A and ML model B are combined through Spearman rank correlation and majority vote to obtain the final material descriptor importance

### Evaluation metrics

The metrics used to evaluate the accuracy of predicted creep rate were Median Absolute Deviation (MAD), Coefficient of Determination ($R^{2}$), Mean Absolute Error (MAE), Root Mean Square Error (RMSE), and Mean Absolute Percentage Error (% Error). Multiple evaluation metrics allow for better understanding of the error produced by ML models (Rengasamy et al. 2019). MAE and RMSE both measure the average magnitude of the error. A larger error is penalised more by RMSE while MAE provides a linear penalty to the magnitude of the error. MAD is a robust measure to outlier in error. $R^{2}$ measures the goodness of fit of predicted output to the actual result. Finally, % Error describes the deviation of error from the actual result in percentage form for more natural understanding.

### Interpreting machine learning model outputs

The results of trained ML models were interpreted in terms of material descriptors importance. The importance value showed how influential each material descriptor is to the final creep rate prediction. The importance values of each material descriptor was calculated using a model-agnostic ensemble material descriptors importance method. The final material descriptors importance was calculated using multiple importance quantification approaches with one or more ML models. The ensemble of multiple material descriptors importance methods lead to more robust and accurate interpretation as opposed to using only one material descriptors importance method (Rengasamy et al. 2020). Ensemble of Permutation Importance (PI) (Fisher et al. 2019) and SHapely Additive exPlanation (SHAP) (Lundberg et al. 2017) between ML models were used to obtain the most important material descriptors in determining the creep rate prediction, as shown in Fig. 10. PI measures the changes in model after one of the material descriptors is randomly shuffled while other material descriptors are unchanged. The process is repeated for each material descriptor. The model’s error remains constant if a reshuffled material descriptor does not contribute to predicting the output. SHAP is a game theory based material descriptor importance method. The importance of each material descriptor is calculated based on its contribution to the predicted output. Contribution of each material descriptor is assigned shapley values using SHAP. Shapely values are based on the average marginal contribution of each material descriptor across possible combinations of all material descriptors. The combinations include adding and removing material descriptors. The material descriptor importance of each ML model obtained through PI and SHAP were fused using Spearman Rank Correlation (SRC) and majority voting (Boyer and Moore 1991). SRC first calculates the pairwise correlation between each material descriptor importance method. Any material descriptor importance method that does not correlate with the other methods were removed as they were considered outlier (Rengasamy et al. 2020). Subsequently, the material descriptors in the remaining methods were ranked based on the majority vote obtained through SRC to produce the final material descriptor importance across all models. The average of majority rank in each material descriptor was calculated to produce the final importance value.

## Results

First, the creep and porosity data, which were used as inputs to the various ML models, will be presented, followed by the ML model results, including material descriptor importance.

### Creep data

**Fig. 11:**
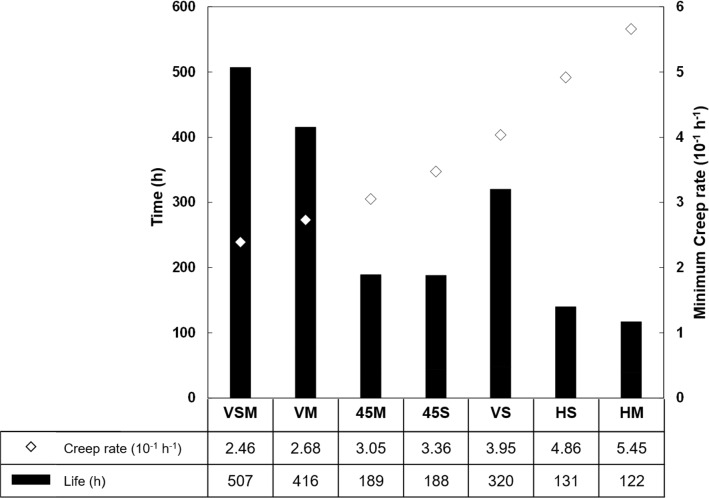
Creep rate and creep life data for the different test cases

Figure 11 shows the relationship between the creep rate and creep life for each test case. VSM had the lowest creep rate, $8 \%$ better than VM, while HM had the highest creep rate. This shows that the creep rate is affected by the build orientation, scan strategy and the number of lasers.

**Fig. 12:**
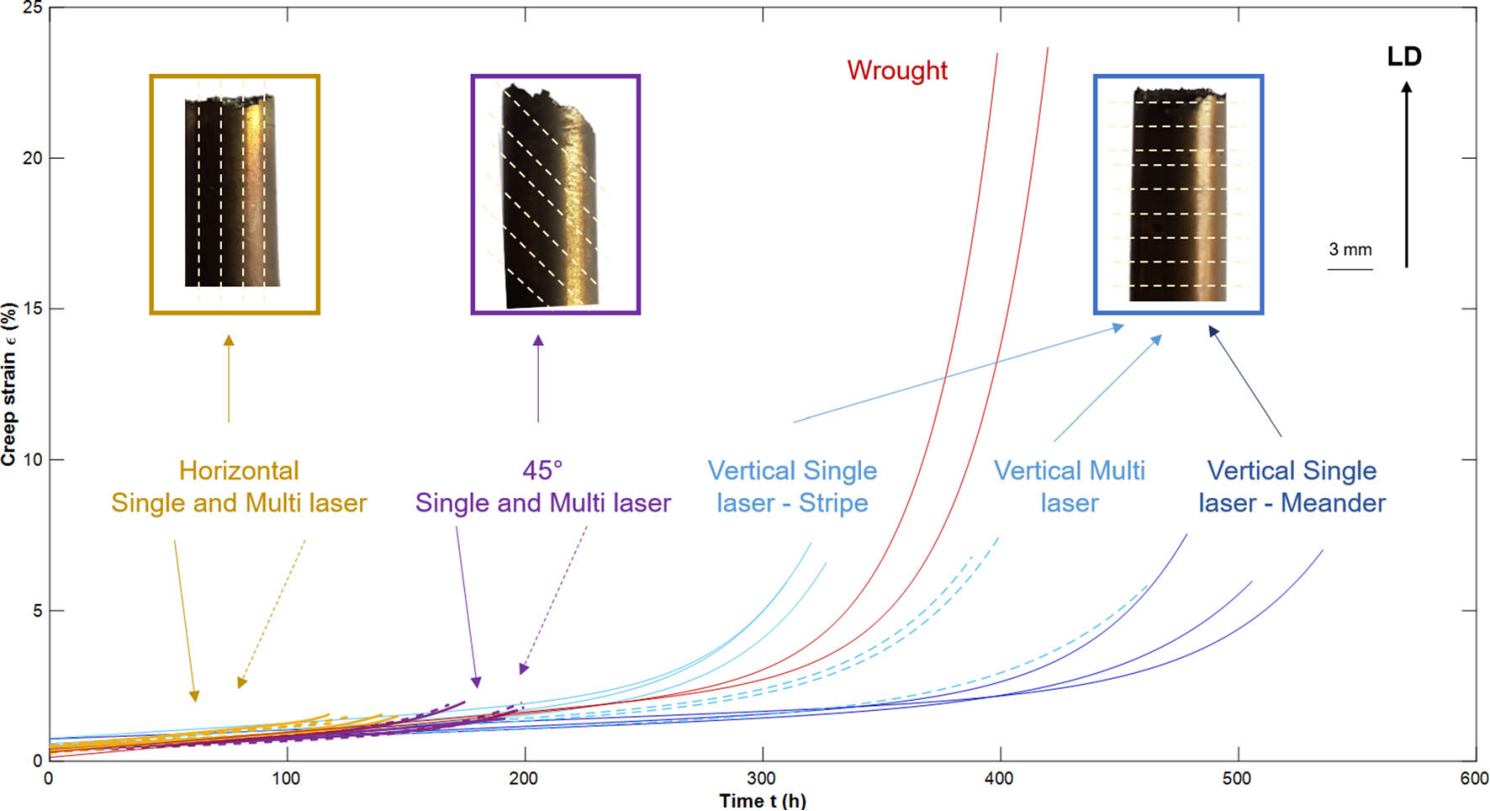
Creep curves for the different samples and compared to Wrought alloy 718

Figure 12 shows the creep curves obtained for the different test cases. The VSM samples had the best creep life, $24 \%$ longer than wrought material, which was included as a benchmark for the rest of the results, whereas LPBF materials usually under perform by over $30\%$ compared to their wrought counterparts, as was observed by Xu et al. (Xu et al. 2018). This shows that specific AM strategies can result in industry standard performance for high temperature creep applications.

Furthermore, Fig. 12 also shows the fracture surfaces of the different samples. It is clear that build orientation affects the fracture mode as the vertical and 45$^\circ$ samples failed on a build layer, perpendicular to their build direction. Whereas the horizontal samples failed at an angle normal to its build layer (i.e. parallel to its build direction). This shows that LPBF build parameters affect the creep performance and failure mechanisms.

### Porosity data

Table 8 presents the porosity data obtained from the image processing programme. VM sample has the highest density, $0.04\%$ different from VSM which has the lowest density. This shows that parts all have a similar density. Other studies have observed both higher densities at $99.99\%$ (Wan et al. 2018) and lower densities (Karabegović 2020), which indicates that the samples have a similar porosity to other LPBF materials. The number of pores varies significantly between samples. The 45M sample has the smallest number of pores, $70\%$ less than VSM.

**Fig. 13:**
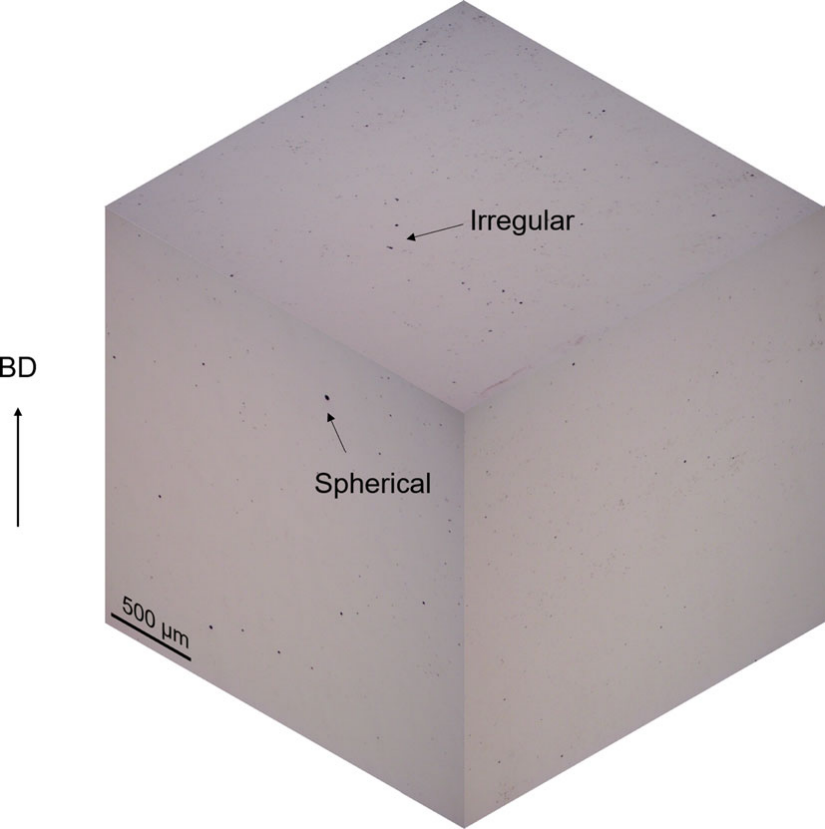
Optical microscope image of the VSM sample in 3 perpendicular planes, showing a mix of spherical and irregular pores

Figure 13 shows the 3-dimensional optical microscope image for the VSM sample, which had the highest porosity. From the figure, there is a majority of spherical pores but some irregular pores can also be observed. Both of these are typical in LPBF material and have been observed in other studies (Deng 2018).

### Machine learning results

The results from RF, GBT, DNN and SVR on the ten-fold cross validation experiment when all test cases were used showed low errors on the predicted creep rates while Ridge and LASSO regressors had high errors as shown in Table 9. RF and GBT performed the best across all evaluation metrics with no error. DNN and SVR had slightly worse performance compared to RF and GBT but were able to learn the data pattern overall. However, DNN had the highest fluctuations after ten repeated experiments. In some cases, such as the MAD and RMSE metrics, the uncertainty in DNN’s result is greater than the averaged predicted value. Despite the larger error of DNN compared to RF and GBT, its performance on $R^{2}$ showed that DNN was able to successfully explain $90\%$ variance in creep rate. Ridge and LASSO regressors were not successful in learning the data pattern and achieved high error suggesting that the data are highly nonlinear. Therefore, only RF and GBT were included in determining the most important material descriptors that affected the predicted creep rate.

**Fig. 14:**
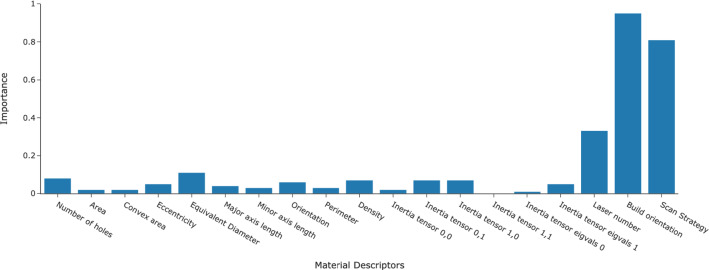
Ensemble material descriptor importance for ten-fold cross validation experiment

Figure 14 shows the ensemble material descriptor importance from RF and GBT. The three most important material descriptors used by the ML models to predict creep rates were the build orientation, scan strategy, and number of lasers. The importance of material descriptors in decreasing order were, build orientation, scan strategy, and number of lasers at $33.0\%$, $28.1\%$, and $11.5\%$ respectively. The three most important material descriptors accounted for $72.6\%$ of importance.

**Table 8:** Summary of porosity data obtained for the different samples investigated

Samples	Density (%)	Number of pores
VSM	99.943	3337
VS	99.947	1841
VM	99.984	1055
45S	99.977	1265
45M	99.980	1002
HS	99.973	1371
HM	99.960	1758

**Table 9:** Creep rate prediction results with 10-fold cross validation

Evaluation metrics	MAD	$R^{2}$	% Error	MAE	RMSE
Random forest	**0.00 **± **0.00**	**1.00** ± **0.00**	**0.00 **± **0.00**	**0.00** ± **0.00**	**0.00 **± **0.00**
Gradient boosted trees	**0.00 **± **0.00**	**1.00 **±** 0.00**	**0.00** ± **0.00**	**0.00 **± **0.00**	**0.00 **± **0.00**
Deep neural network	0.44 ± 0.58	0.90 ± 0.11	30.45 ± 45.53	2.24 ± 1.90	4.98 ± 5.23
SVR	0.56 ± 0.13	0.86 ± 0.09	5.07 ± 0.31	2.37 ± 0.12	4.39 ± 0.14
Ridge regressor	7.48 ± 0.15	0.42 ± 0.04	20.65 ± 0.78	7.68 ± 0.23	8.99 ± 0.28
LASSO regressor	8.09 ± 0.23	0.39 ± 0.02	20.22 ± 0.46	7.73 ± 0.18	9.18 ± 0.15

All numbers are in $1\mathrm {e}{-4}$ except for $R^{2}$ and % Error

**Fig. 15:**
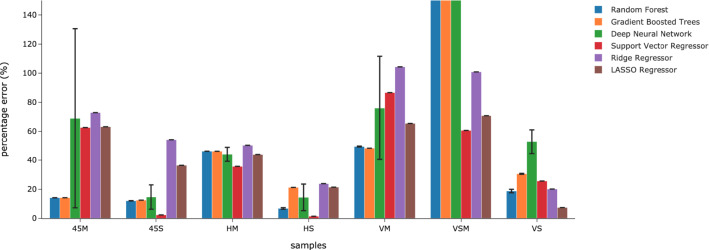
Percentage error of creep rate predictions for Leave-One-Condition-Out experiment using Random Forest, Gradient Boosted Trees, Deep Neural Network, Support Vector Regressor, Ridge Regressor, and LASSO Regressor. The Y-axis shown here is limited to the range of 0-150% to provide a better view as the percentage error for VSM predicted by Random Forest, Gradient Boosted Trees, and Deep Neural Network are greater than 400%

Subsequently, the LOCO experiment was conducted and the results are shown in the Fig. 15 and Table 10. The best results for each left out conditions were made bold in the Table. RF performed the best in predicting the creep rate of VS test cases when they were left out of the training data. GBT achieved the best creep rate prediction for 45M and VM test cases while SVM predicted 45S, HM, HS, and VSM creep rate with the lowest error. Overall, the creep rate prediction for 45M, 45S, HS, and VS had the lowest error at less than $20\%$ error for all as indicated by the % Error evaluation metric. The predicted creep rate for HS achieved the lowest % Error at $1.40\%$. Predicted VM and HM creep rate % Error were higher compared to 45M, 45S, HS, and VS at $48.14\%$ and $35.80\%$ respectively. The highest predicted error was for the VSM test case. The prediction for VSM creep rate was considered non-predictive by RF, GBT and DNN as the % Error error were greater than $400\%$ for each model but SVR were able to narrow the error down to $60.68\%$. Furthermore, the result of LASSO Regressor had low error for VS creep rate prediction. However, further investigation showed that the LASSO model had predicted the average value of all creep rates which coincidentally is very close to the VS creep rate resulting in low error but the model itself did not learn the data pattern. Between all the models tested, DNN had the highest uncertainty in its prediction while RF GBT, SVR, Ridge Regressor, and LASSO Regressor prediction had low uncertainty throughout ten repeated experiments.

**Fig. 16:**
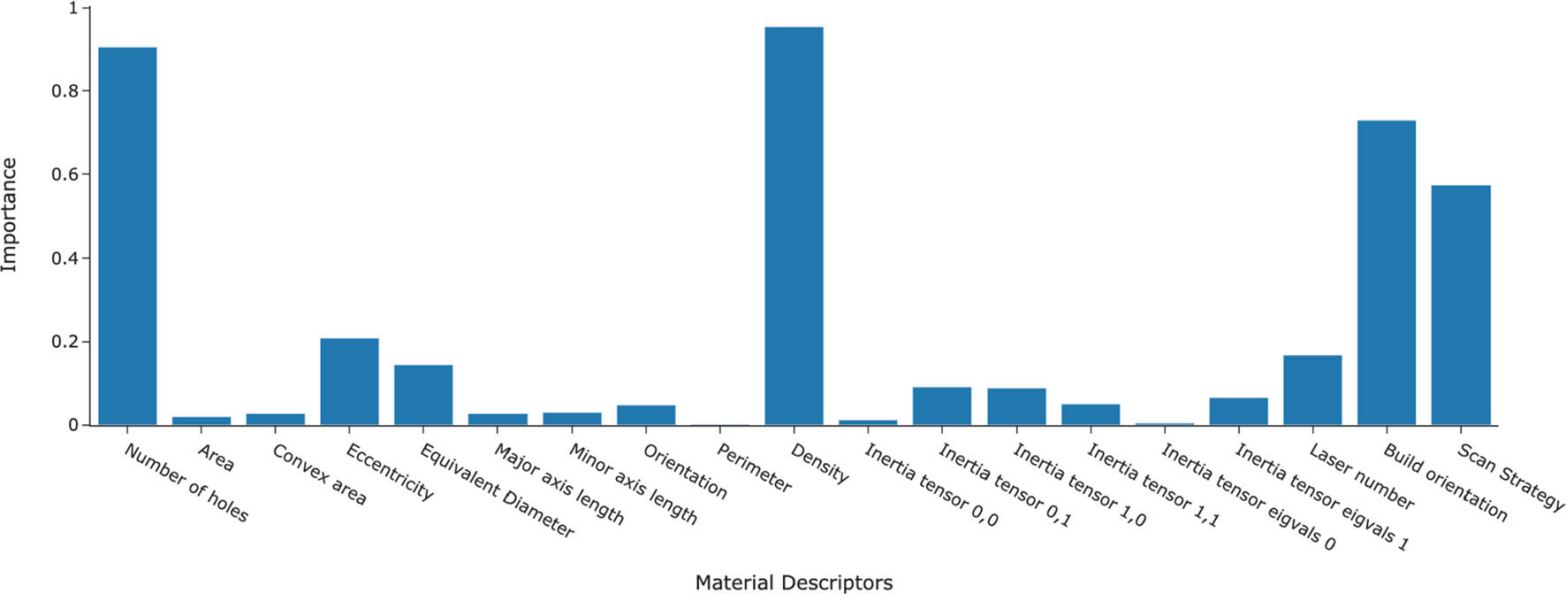
Ensemble material descriptor importance for Leave-One-Case-Out experiment

Figure 16 shows the ensemble material descriptor importance from top three most performant ML models i.e. RF, GBT, and SVM for the LOCO experiment. The four most important material descriptors used by the three ML models to predict creep rates were the density, number of pores, build orientation, and scan strategy in decreasing order. The importance of material descriptors for density, number of pores, build orientation, and scan strategy were $23.0\%$, $21.9\%$, $17.7\%$, and $13.8\%$ respectively. The four most important material descriptors accounted for $76.4\%$ of importance. The remaining fifteen material descriptors were considered less important and had low contributing factor as they only accounted for $23.6\%$ of material descriptor importance.

## Discussion

### Material descriptors affecting the creep rate

Results from Fig. 16 showed that the top factors influencing the creep rate were the density, number of pores, build orientation and scan strategy, from most to least important, respectively.

#### Effects of density

Two types of pores, typical in LPBF, were observed in the samples: spherical pores, formed from gas entrapment, and irregular pores, which can serve as crack initiation points and lead to failure (Deng 2018). When creep occurs, voids form on the grain boundaries or in high stress concentration areas such as irregular pores, Laves phase or carbides. Hence, it is logical that the number of pores, particularly the number of irregular pores, would affect the density and significantly impact the creep rate. One study, which looked at the defect evolution of LPBF alloy 718 during creep, found that the number and size of pores and defects increase with time as the material is creeping (Xu et al. 2017b). This means that the low creep rate of the 45M samples could be due to the low number of pores present compared to other samples. By tracking defects and pores, the prediction of time-dependent failure is possible for creep (Xu et al. 2017a) and Fatigue (Sheridan et al. 2018). Furthermore, some damage mechanics models, such as the Kachanov creep-damage model, use the presence of pores and defects (i.e. damage) in order to predict the creep rate of materials (Hyde 2014). This shows that density and the number of pores can definitely be used as an input to ML models in order to predict the minimum creep rate. However, the density and the number of pores in LPBF samples are caused by the LPBF process itself, from the different build parameters.

**Table 10:** Creep rate prediction results for Leave-One-Condition-Out experiment

Models	Evaluation metrics	45M	45S	HM	HS	VM	VSM	VS
Random forest	MAD	4.31 ± 0.01	4.24 ± 0.02	26.09 ± 0.00	1.55 ± 0.41	13.42 ± 0.08	100.10 ± 0.00	**7.50** ± **0.76**
	MAE	4.32 ± 0.01	4.19 ± 0.03	26.07 ± 0.02	3.33 ± 0.27	13.46 ± 0.07	100.10 ± 0.00	**7.59** ± **0.49**
	% Error	14.17 ± 0.04	12.05 ± 0.11	46.06 ± 0.04	6.78 ± 0.55	49.32 ± 0.28	418.82 ± 0.00	**18.80** ±** 1.21**
	RMSE	4.32 ± 0.01	4.19 ± 0.03	26.07 ± 0.02	4.12 ± 0.27	13.46 ± 0.07	100.10 ± 0.00	**8.75 **±** 0.31**
Gradient boosted trees	MAD	**4.31 **± **0.00**	4.28 ± 0.00	26.08 ± 0.00	7.41 ± 0.00	**13.17 **± **0.00**	100.09 ± 0.00	13.17 ± 0.07
	MAE	**4.31** ± **0.00**	4.35 ± 0.01	26.05 ± 0.01	10.47 ± 0.02	**13.14 **± **0.00**	100.09 ± 0.00	12.39 ± 0.13
	% Error	**14.19** ± **0.02**	12.50 ± 0.02	46.02 ± 0.01	21.29 ± 0.04	**48.14 **± **0.01**	418.82 ± 0.00	30.67 ± 0.32
	RMSE	**4.32** ± **0.01**	4.35 ± 0.01	26.05 ± 0.01	10.99 ± 0.02	**13.14 **± **0.00**	100.09 ± 0.00	12.52 ± 0.11
Deep neural network	MAD	20.96 ± 18.92	4.69 ± 3.16	25.39 ± 2.46	6.73 ± 5.10	20.07 ± 10.68	99.86 ± 0.18	14.62 ± 5.73
	MAE	21.02 ± 18.92	5.10 ± 3.16	24.88 ± 2.46	7.10 ± 5.10	20.76 ± 10.68	98.55 ± 0.18	21.30 ± 5.73
	% Error	68.94 ± 61.69	14.65 ± 8.40	43.96 ± 4.80	14.43 ± 9.16	76.06 ± 35.55	412.38 ± 2.66	52.73 ± 8.30
	RMSE	21.40 ± 18.5	5.69 ± 2.70	35.10 ± 2.79	7.97 ± 4.31	21.97 ± 10.37	98.62 ± 0.59	27.70 ± 4.91
SVR	MAD	19.33 ± 0.00	**0.75** ± **0.00**	**20.12** ± **0.00**	**0.51** ± **0.00**	23.68 ± 0.00	**14.64** ± **0.00**	10.26 ± 0.00
	MAE	19.11 ± 0.00	**0.81** ±** 0.00**	**20.26** ± **0.00**	**0.69 **± **0.00**	23.68 ± 0.00	**14.50** ± **0.00**	10.37 ± 0.00
	% Error	62.64 ± 0.00	**2.34** ±** 0.00**	**35.80** ± **0.00**	**1.40 **± **0.00**	86.74 ± 0.00	**60.68** ± **0.00**	25.69 ± 0.00
	RMSE	19.12 ± 0.00	**0.93** ± **0.00**	**20.26** ± **0.00**	**0.91** ± **0.00**	23.68 ± 0.00	**14.51** ± **0.00**	10.41 ± 0.00
Ridge regressor	MAD	22.29 ± 0.00	19.23 ± 0.00	28.20 ± 0.00	12.06 ± 0.00	28.44 ± 0.00	22.67 ± 0.00	4.22 ± 0.00
	MAE	22.25 ± 0.00	18.87 ± 0.00	28.39 ± 0.00	11.75 ± 0.00	28.49 ± 0.00	24.11 ± 0.00	8.15 ± 0.00
	% Error	72.96 ± 0.00	54.22 ± 0.00	50.17 ± 0.00	23.89 ± 0.00	104.36 ± 0.00	100.89 ± 0.00	20.17 ± 0.00
	RMSE	22.26 ± 0.00	18.95 ± 0.00	28.40 ± 0.00	11.89 ± 0.00	28.49 ± 0.00	24.90 ± 0.00	11.51 ± 0.00
LASSO regressor	MAD	19.27± 0.00	12.71 ± 0.00	24.80 ± 0.00	10.57 ± 0.00	17.88 ± 0.00	16.92 ± 0.00	2.99 ± 0.00
	MAE	19.27 ± 0.00	12.71 ± 0.00	24.80 ± 0.00	10.57 ± 0.00	17.88 ± 0.00	16.92 ± 0.00	2.99 ± 0.00
	% Error	63.20 ± 0.00	36.52 ± 0.00	43.82 ± 0.00	21.48 ± 0.00	65.51 ± 0.00	70.82 ± 0.00	7.41 ± 0.00
	RMSE	19.27 ± 0.00	12.71 ± 0.00	24.80 ± 0.00	10.57 ± 0.00	17.88 ± 0.00	16.92 ± 0.00	2.99 ± 0.00

All numbers are in $1\mathrm {e}{-4}$ except % error

#### Effects of LPBF build parameters

Out of the top material descriptors affecting the creep rate, build orientation and scan strategy were present, while the number of lasers only seemed to have a minor effect. This section will show the effect of the build parameters on the density and number of pores as well as the effect on the creep rate.

***Effect on Density***

From Table 8, it seems that the the scan strategy and the build orientation do not have a significant effect on the part density. Although no studies have related build orientation to porosity, scan strategies are known to have an effect. Mancisidor et al. (2016) found residual porosity to be mostly present in scan strategies such as the Stripe strategy, due to excessive energy density in laser overlap regions. However, in the present work, the difference in density between the Stripe and Meander strategies was inferior to $4\times 10^{-3} \%$, clearly showing that the Stripe strategy did not result in a significantly higher porosity. It should be noted that, despite an equivalent density, the Meander Strategy has 81% more pores than the Stripe strategy. Moussaoui et al. (2018) stated that porosity reduces when the Volumetric Energy Density increases. Hence, since there are less laser overlap zones, which receive increased heat input, in the Meander strategy, pores could have been more likely to form than for samples with the Stripe strategy. However, an excessive heat input was also found to result in an increase in spherical pores ( Zhang et al. 2020). Hence, there is some disagreement between studies and more experimentation should be done. Furthermore, the porosity analysis was performed using cubes, while the creep specimen were cylinders. Some studies on Electron Beam Powder Bed Fusion of various metals have found that the sample geometry affects the porosity (Frederick et al. 2018; McNeil et al. 2020), pore location (Yoder et al. 2019) and mechanical properties (Yoder et al. 2018). However, these studies compared simple (e.g. bulk) and complex (e.g. topologically optimised designs and nested geometries) Electron Beam Powder Bed Fusion built geometries, while the cubes and cylinders built in this work are both bulk material with similar surface area dimensions. Hence, the correlation between cube and cylinder geometries for LPBF material is assumed with confidence.

Therefore, it is clear that although orientation does not seem to have an effect on the sample density, a review of the literature showed that other AM build parameters, such as the scan strategy, laser energy density, scanning speed (Choi et al. 2017; Kumar et al. 2019; Xia et al. 2017) and others have an effect on the porosity and number of pores. Porosity, in turn is one of the material descriptors that most affects the creep rate. Therefore, it would be useful to develop a ML model where more LPBF build parameters would be included as inputs to obtain the material density as an output. This would positively impact AM as it would save a significant amount of time and money usually spent in process optimisation. Additionally, Neural Networks have been used in Selective Laser Sintering to predict component density by using laser power, scan speed, layer thickness, stripe offset (Shen et al. 2004) as well as the hatch spacing, scan mode, temperature and interval time (Wang et al. 2009).

***Effect on Creep***

As aforementioned, the scan strategy could be contributing to the difference in density in the sample, which affects the creep performance.The microstructure resulting from the use of different scan strategies has also been shown to affect creep behaviour (Sanchez et al. 2021a).

The build orientation has also been shown to affect the creep rate (Sanchez et al. 2021a), due to the resulting microstructure it produces. It is common knowledge that LPBF microstructure results in elongated grains in the Build Direction (Amato et al. 2012). This means that for the different build orientations, the elongated grains were angled differently, with respect to the loading direction. From Figs. 11 and 12, it is clear that the build orientation affects the creep rate and the creep life. This is because creep failure occurs by void coalescence on grain boundaries and those boundaries are at different angles, resulting in different modes of failure. Figure 12 shows the fracture surface where the 45$^\circ$ sample failed on a build layer, at 45$^\circ$ angle from the loading direction. The vertical samples behaved in a similar fashion while horizontal samples failed normal to their build layer. This was also observed in creep testing of alloy 718 (Kuo et al. 2017) and high temperature tensile testing (Hilaire et al. 2019), where Horizontal samples performed worse than their Vertical counterparts (Kuo et al. 2017).

The number of lasers per part, as predicted by this model, plays a less significant role than build orientation on the creep behaviour, which confirms the results obtained in previous studies (Sanchez et al. 2021b). Figure 12 shows that there are no differences between the creep life of multi laser and single laser for Horizontal and 45$^\circ$ samples, whereas there is a difference for the vertical samples. This shows that grain orientation is a more determinant factor on the creep rate than the number of lasers. As LPBF build parameters were found to be some of the most influencing material descriptors for the creep rate by the ML Model, more research should be done in this area. A different model taking into account other LPBF parameters—such as laser power, hatch spacing and others — could be developed in order to further understand their effect on the creep performance.

### Interpretation of machine learning models’ predictions

The ML models showed that they were able to accurately predict the creep rate of a limited materials testing data set, shown in Table 9. When all test cases were used to train the models. RF and GB were able to predict the creep rate to $0\%$ Error consistently because the distribution of dataset in the training and testing set were similar across all cross-validation. DNN and SVR had slightly higher errors in the creep rate prediction than RF and GBT while Ridge and LASSO Regressors failed to predict the creep rate accurately. DNN was the only ML model that was highly uncertain on the predicted creep rate. The high error from DNN could be the result of overfitting of the model through low number of training instances. Overfitting of models resulted in poor generalisation of model across all ten-fold of cross validation. As the model parameters far exceeds the number of instances it began to memorise the training dataset (Zhang et al. 2018) Furthermore, DNN requires a large and diverse training instances to achieve generalisation of model. On the other hand, the reason why the Ridge and LASSO Regressors failed to predict the creep rate was due to the fact that they are linear models and the relationship between material descriptors and creep rate are non-linear. In the subsequent LOCO experiment, DNN experienced similar difficulty in generalising the model for all test cases. Similarly, Ridge and LASSO Regressors failed to map the relationship between material descriptors and creep rate. RF, GBT, and SVR were able learn the data pattern for 45M, 45S, HS, and VS when they were left out of the training set. When test cases share similar print conditions but have different creep rate e.g. HS and HM, the ML models attempts to generalise both test cases together which leads to high errors. Additionally, some combinations of test cases in the training data might provide more vital information to assist ML models to generalise better.

The ensemble material descriptor importance obtained from the ML models was able to accurately identify the most important material descriptors affecting creep rate. Using ML models and interpretable methods allowed information such as important material descriptors that would otherwise have been difficult to obtain using traditional methods or more extensive experimentation. Although FEA may result in slightly more accurate creep rate prediction, it is unable to give an explanation of which factors causes these fluctuations in the manufacturing process. Additionally, the adaptation of FEA models for AM specific characteristics, such as build parameters and porous microstructure, has yet to be addressed.

Therefore, ML is not discounted as a powerful tool for AM. Multiple build parameters can be included in ML models, unlike FEA. These inputs can be used for density prediction, mechanical property prediction and more. One downside of ML is that most models require a lot of data. The more data available to train the model, the more accurate that model will be. Indeed, predicting the creep rate using LOCO was obtained from a population size of 512 images whereas most ML models have a population size in the hundreds of thousands, resulting in extremely accurate predictions. By inputting more experimental data, the models’ accuracy should improve (Bustillo et al. 2020). Thus, the findings here are limited to the small available data as it is difficult to generate large creep dataset, and should be further validated in the future, as more data is made available. Additionally, it is difficult to empirically select which ML model or set of ML models in the case of ensemble will perform well in its prediction task before starting the experiment. Therefore a lot of trial and error or reliance on heuristic and experience is necessary to select the right set of ML models.

As experimentation is costly and time consuming, a potential way around the big data set required by ML would be to use data from other research institutions, published in papers. Since process parameters can be used as inputs for the models, data from other papers and researchers could potentially be used by inputting differentiating parameters such as powder and machine used to fabricate parts as well as build parameters -and of course, the corresponding creep rate, density or other parameter of interest. Additionally, ML models are less computationally expensive than FEA models and hence can allow for a quicker turnover and numerous experiments.

## Conclusions and future work

This work aimed at predicting the creep rate of LPBF samples by using a ML model and to determine the main LPBF factors affecting the creep rate. Some contributions from this work include:

- Various ML models were able to predict the creep rate using porosity data and LPBF build parameters despite a limited amount of data.
- Random Forest and Gradient Boosted Trees were able to accurately predict the creep rate with 100% accuracy when all test cases were used to train the model. Support Vector Regressor achieved the 98.6% accuracy when one case was left out at its best.
- The top material descriptors affecting the creep rate were identified to be density, number of pores and build orientation, in descending order of importance. The number of lasers was found to have only an insignificant effect on the creep rate.
- Density and the number of pores serve as crack initiation points and negatively affect the creep rate; the scan strategy was found to affect component density and the build orientation affected the failure of samples.
- The main disadvantage of using ML models is the need for large sample size in order to increase accuracy. However, accurate ML models were trained by using data augmentation to increase sample size and image analysis to extract material descriptors. But this could be solved by more collaboration between researchers.
- Finally, despite using a small data set, some ML models were able to offer insight into the effect of process parameters on creep properties as well as to predict creep rates.

Overall, the ability to predict the behaviour and life of engineering components has always been essential for those components to be used in critical applications. AM of critical components has great potential but the complex relationship between print parameters are still to be understood. By being able to predict the creep rate of an AM nickel-based superalloy as well as identifying the most critical print parameters, this approach gives an insight into the significant impact that combining AM (or other manufacturing processes) with ML can have. Future work includes developing new ML models which could use the LPBF process parameters as inputs and output the part density, creep rate and other properties. This would allow to gain a better understanding of these complex relationships. Combining FEA and ML could be another potential way to make use of this technology for AM. Additionally, active learning can be incorporated as part of the ML learning process to utilise human intervention to combat low training sample size.

Finally, this paper shows the potential of combining manufacturing processes and ML to understand the effect of process parameters on mechanical properties.
